# Low expression of GSTP1 in the aqueous humour of patients with primary open‐angle glaucoma

**DOI:** 10.1111/jcmm.16361

**Published:** 2021-02-17

**Authors:** Aihua Liu, Liming Wang, Qiang Feng, Dandan Zhang, Kexi Chen, Guli Humaer Yiming, Qiong Wang, Yaru Hong, Amy Whelchel, Xiaomin Zhang, Xiaorong Li, Lijie Dong

**Affiliations:** ^1^ Tianjin Key Laboratory of Retinal Functions and Diseases Tianjin International Joint Research and Development Centre of Ophthalmology and Vision Science Eye Institute and School of Optometry Tianjin Medical University Eye Hospital Tianjin China; ^2^ Ophthalmology Department of People's Hospital of Hotan District Xinjiang China; ^3^ Department of Physiology University of Oklahoma Health Sciences Center Oklahoma OK USA

**Keywords:** aqueous humour, cataract, GSTP1, inflammation, oxidative stress, primary open‐angle glaucoma, proteome

## Abstract

Primary open‐angle glaucoma (POAG) is characterized by irreversible neurodegeneration accompanied by visual field defects and high intraocular pressure. Currently, an effective treatment is not available to prevent the progression of POAG, other than treatments to decrease the high intraocular pressure. We performed proteomic analysis of aqueous humour (AH) samples from patients with POAG combined with cataract and patients with cataract to obtain a better understanding of the pathogenesis of POAG and explore potential treatment targets for this condition. Samples were collected from 10 patients with POAG combined with cataract and 10 patients with cataract. Samples from each group were pooled. A high‐resolution, label‐free, liquid chromatography‐tandem mass spectrometry‐based quantitative proteomic analysis was performed. In total, 610 proteins were identified in human AH samples from the two groups. A total of 48 up‐regulated proteins and 49 down‐regulated proteins were identified in the POAG combined with cataract group compared with the control group. Gene Ontology (GO) analysis revealed key roles for these proteins in inflammation, immune responses, growth and development, cellular movement and vesicle‐mediated transport in the biological process category. Kyoto Encyclopedia of Genes and Genomes (KEGG) analysis indicated the down‐regulated expression of glutathione S‐transferase P (GSTP1) in the glutathione metabolism signalling pathway in the POAG combined with cataract group. Additionally, certain significantly differentially expressed proteins in the proteomic profile were verified by enzyme‐linked immunosorbent assay (ELISA). GSTP1 levels were reduced in the human AH samples from the POAG combined with cataract group, based on the results of ELISA and proteomic profiling. Therefore, GSTP1, a redox‐related marker, may be involved in the pathological process of POAG and may become a treatment target in the future.

## 1 INTRODUCTION

Primary open‐angle glaucoma (POAG) is defined as a chronic progressive optic neuropathy associated with elevated intraocular pressure (IOP) and visual field defects, and this disorder is one of the leading causes of irreversible blindness worldwide.^1^ The dynamic balance of IOP is closely related to drainage of the aqueous humour (AH). A high IOP occurs as the result of impaired AH outflow.^2^, ^3^ The AH performs various functions, including maintaining IOP, providing nutrients to the ocular tissues and removing metabolic products from ocular tissues.^4^, ^5^ Changes in the protein composition of the AH are closely related to the metabolism of the anterior segmental tissue, such as the trabecular meshwork (TM).^6^

The TM acts as a static filter that exerts stable resistance to AH outflow.^7^ Abnormal expression of extracellular matrix (ECM) components within the TM microenvironment is associated with impaired AH drainage and leads to an elevated IOP.^8^ In addition, abnormal expression of ECM proteins in the TM is related to the oxidative stress response in the TM.^9^ Many ocular diseases and related treatments are associated with oxidation and inflammation.^10^, ^11^, ^12^, ^13^, ^14^, ^15^ Some studies have also reported markedly elevated levels of oxidative stress markers in the AH from patients with POAG, along with altered expression of markers of antioxidant defences and an enhanced inflammatory response.^16^, ^17^, ^18^

Quantitative proteomics based on mass spectrometry (MS) is an important methodology for biological and clinical research.^19^, ^20^, ^21^, ^22^ Label‐free MS has several advantages, including the lack of a restriction on the sample size, the lack of a requirement for expensive isotope labelling and the ability to detect a wide range of proteins.^23^ Comparison and identification of the changes in proteins in the AH using proteomics help researchers detect differentially expressed proteins in patients with different diseases. In this study, we sought to detect differentially expressed proteins and explore the pathological mechanisms of POAG, which will provide new molecular targets for glaucoma treatment.

## 2 MATERIALS AND METHODS

### 2.1 Subjects

**FIGURE 1:**
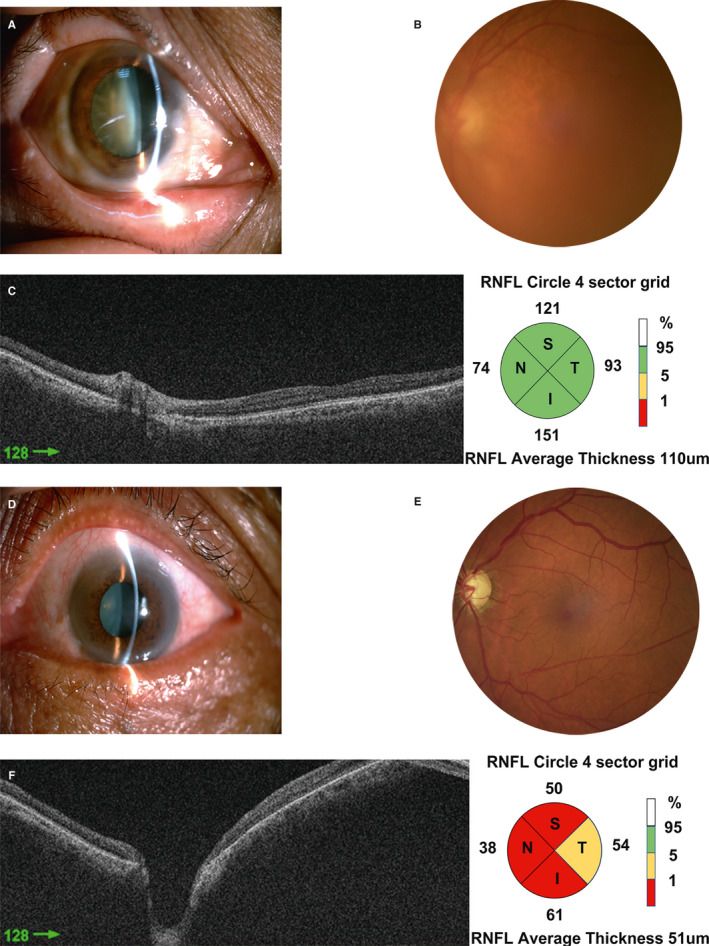
Patients’ diagnostic examinations. Patients with cataract have a turbid lens, normal anterior chamber depth (A), formal cup/disc ratio (B) and regular structure of the optic nerve head (C). However, patients with POAG combined with cataract display slight turbidity of the lens, a normal anterior chamber depth (D), abnormal cup/disc ratio (E) and irregular optic nerve head (F)

The Institutional Ethics Committee approved this study, and we adhered to the tenets of the Declaration of Helsinki when conducting experiments involving human subjects. Patients were enrolled after providing informed consent at the Tian Medical University Eye Hospital. Samples were collected at the Tianjin Medical University Eye Hospital from October 2016 to December 2017. Twenty subjects, including 10 patients with POAG combined with cataract and 10 age ‐and sex‐matched patients with cataract as a control group, were recruited for this study. All subjects underwent a thorough ophthalmic evaluation by a glaucoma specialist using standard diagnostic criteria, including glaucoma‐related measurements such as IOP, visual acuity, gonioscopy examination, fundus photography, preoperative cup‐disc ratio and Humphrey visual field analysis (Figure 1). The following inclusion criteria were used for patients with POAG combined with cataract: IOP > 21 mm Hg (1 mm Hg = 0.133 kPa), expanding cup/disc, narrowing visual field and open anterior chamber angle. The patients were also diagnosed with cataract. The criteria for cataract included an opaque lens and a progressive decrease in visual acuity. None of the control subjects had a history of glaucoma. Patients with ophthalmic conditions such as uveitis, ocular trauma; a history of ocular surgery within the previous three months; intraocular inflammation; secondary or neovascular glaucoma; or use of topical or systemic corticosteroids were excluded from the study.

AH samples (25‐100 μL) were collected through an anterior chamber paracentesis using a 30‐gauge needle at the beginning of the surgical procedures from patients undergoing cataract or trabeculectomy surgery. Samples were transported on ice, centrifuged at 704 *g* for 10 minutes at 4°C and immediately stored at −80°C until further analysis.

### 2.2 Estimation of the total protein content using the Bradford method

The total protein concentration in the AH was determined using a Bradford assay kit (Thermo Fisher Scientific) according to the manufacturer's protocol. Briefly, 5 μL of AH proteins was added to 200 μL of Bradford reagent. The optical density was measured at 595 nm after a 15 minutes incubation. The protein concentration of each sample was calculated from a standard curve using BSA as the reference.

### 2.3 Proteolysis

Approximately 5 μg of total AH protein was aliquoted, the volume was brought to 500 μL with 5 mmol/L ammonium bicarbonate (NH_4_HCO_3_), and the mixture was concentrated using a 3 kD molecular weight cut‐off filtration column. Next, a solution containing 100 mmol/L dithiothreitol (DTT) and 50 mmol/L NH_4_HCO_3_ was added to the solution and incubated at 60°C for 30 minutes. Subsequently, a solution of 200 mmol/L iodoacetamide and 50 mmol/L NH_4_HCO_3_ was added and incubated with the sample in the dark at 37°C for 20 minutes. Trypsin was added to the solution at a 1:50 dilution and incubated at 37°C overnight, after which it was further incubated at 37°C for 20 minutes along with formic acid. Samples were centrifuged at 15 339 *g* for 10 minutes to remove the debris, and the supernatant was filtered through a 0.2 μm filter and dried in a speed vacuum. Then, the dried residues in the vials were reconstituted with 2% acetonitrile and 0.1% formic acid, centrifuged, and the supernatant was transferred into total recovery vials. Trypsin‐digested AH proteins from each group (n = 10) of samples were subjected to liquid chromatography‐tandem mass spectrometry (LC‐MS/MS) analysis.

### 2.4 Nano‐HPLC‐MS (Q‐Exactive) proteomic and data analyses

Samples were subjected to MS analysis (Thermo Fisher). Components obtained by high pH reverse‐phase chromatography were resolved with 20 μL of 2% methanol and 0.1% formic acid. Samples were centrifuged at 11 269 *g* for 10 minutes. Then, 10 μL of the supernatant was loaded using the sandwich method. The loading pump flow rate was 350 nL/min for 15 minutes, and the separation flow rate was 350 nL/min. The following separation gradient was used: phase B percentage (%) 4/0 min, 15/5 min, 25/40 min, 35/65 min, 95/70 min, 95/82 min, 4/85 min and 4/90 min. The following ion source parameters were used: spray voltage of 2.1 kV and capillary temperature of 250°C. The full MS were obtained at a resolution of 70 000 FWHM and full scan AGC target of 1e6. The dd‐MS2 data were obtained at a resolution of 17 500 FWHM and AGC target of 5e6. The label‐free MS analysis was performed using a mass spectrometer, and the raw MS data were processed using MaxQuant software.

### 2.5 Quantification of significantly differentially expressed proteins using enzyme‐linked immunosorbent assay (ELISA)

AH samples from control patients with cataract alone (n = 21) and patients with POAG combined with cataract (n = 20) were collected to determine the levels of the significantly differentially expressed proteins glutathione S‐transferase P (GSTP1), C‐reactive protein (CRP), procollagen‐lysine, 2‐oxoglutarate 5‐dioxygenase 1 (PLOD1), transforming growth factor ß (TGF‐ß), growth differentiation factor 11 (GDF11) and tenascin (TNC) using ELISA kits (mlbio). Each individual sample was used independently and individually. The procedure described below was performed according to the manufacturer's protocol. Preparation: The collected AH samples were removed from the −80°C freezer, dissolved at room temperature and centrifuged at 704 *g* for 30 min. Standard wells and sample wells were established. Each standard well was filled with 50 μL of the standards. Forty microlitres of the sample dilution solution and 10 μL of the sample solution (fivefold final diluted concentration of the sample) were added to each of the sample wells. The samples were gently shaken. One hundred microlitres of enzyme‐labelled reagent was added to each well except for the blank wells. The plate was sealed with a membrane and incubated at 37°C for 60 minutes. The plate was washed with X1 washing solution and incubated for 30 seconds, the liquid was discarded, and the plate was dried. This procedure was repeated five times. Fifty microlitres of developer‐A was added to each well, followed by 50 μL of developer‐B. The samples were gently shaken and incubated at 37°C in the dark for 15 minutes. Fifty microlitres of stop solution was added to each well to stop the reaction. The absorbance (OD value) of each well was measured at a wavelength of 450 nm over 15 minutes. The linear regression equation of the standard curve was calculated using the concentration of the standard and the OD values. The OD value of the sample was input into the equation, the sample concentration was calculated, and then the value was multiplied by the dilution factor of 5 to obtain the actual concentration of the sample.

### 2.6 Statistical analysis

Protein expression profiles were analysed with MaxQuant software (version 1.6.2.0.). MaxQuant significance A^24^ was used to evaluate the significance of differences. Differentially expressed proteins were identified from the raw data (fold change > 1.5 and *P* < .05). The data were processed and analysed using GraphPad^®^ Prism 7 software. Clinical variables and ELISA data were analysed using an unpaired *t* test followed by the Mann‐Whitney *U* test. Data are presented as means ± SDs, and *P* < .05 was considered to indicate a statistically significant difference.

## 3 RESULTS

### 3.1 Patient information

According to the inclusion and exclusion criteria, patients with cataract had a turbid lens, normal anterior chamber depth, appropriate cup/disc ratio and regular structure of the optic nerve head. Patients with POAG combined with cataract displayed slight turbidity of the lens and a normal anterior chamber depth, abnormal cup/disc ratio and irregular optic nerve head. The present study included 10 eyes from 10 patients with POAG combined with cataract and 10 eyes from 10 patients with cataract. The demographic and clinical characteristics are summarized in Table 1. The mean ages of the patients with POAG combined with cataract and the controls were 72.8 ± 2.6 years and 71.7 ± 2.5 years, respectively (*P* = .923). No significant differences in the sex distribution, axial length, corneal thickness, aqueous depth or visual acuity were observed between the two groups (*P* > .05). As expected, the POAG combined with cataract group had a higher mean IOP and larger cup/disc ratio than the control group (*P* = .043 and *P* = .0005, respectively).

**TABLE 1:** Demographic and clinical characteristics of Cataract, POAG combined cataract Subjects

Characteristics	POAG	Cataract	*P*‐value	Significance
Subjects, n	10	10		
Male/female	8/2	8/2	.9999	ns
Age, y (mean ± SD)	72.80 ± 2.60	71.70 ± 2.51	.7644	ns
Cup/disc ratio (mean ± SD)	0.62 ± 0.06	0.36 ± 0.02	.0005	***
IOP (mean ± SD), mm Hg	30.50 ± 0.90	15.34 ± 0.77	<.0001	****
Axial length (mean ± SD), mm	23.94 ± 0.50	23.66 ± 0.23	.6243	ns
Corneal thickness (mean ± SD), µm	530.80 ± 13.53	520.50 ± 6.49	.5012	ns
ACD (mean ± SD), mm	3.03 ± 0.09	3.09 ± 0.11	.6636	ns
BCVA (mean ± SD)	0.50 ± 0.09	0.31 ± 0.07	.1153	ns
Other disease history	–	–	–	

Note

Statistical analysis: nonparametric *t* test (*** represents *P* < .001; **** represents *P* < .0001; ns represents no significant difference).

Abbreviations: ACD, anterior chamber depth; BCVA, best corrected visual acuity; IOP, intraocular pressure; SD, standard deviation.

### 3.2 Data acquisition

**FIGURE 2:**
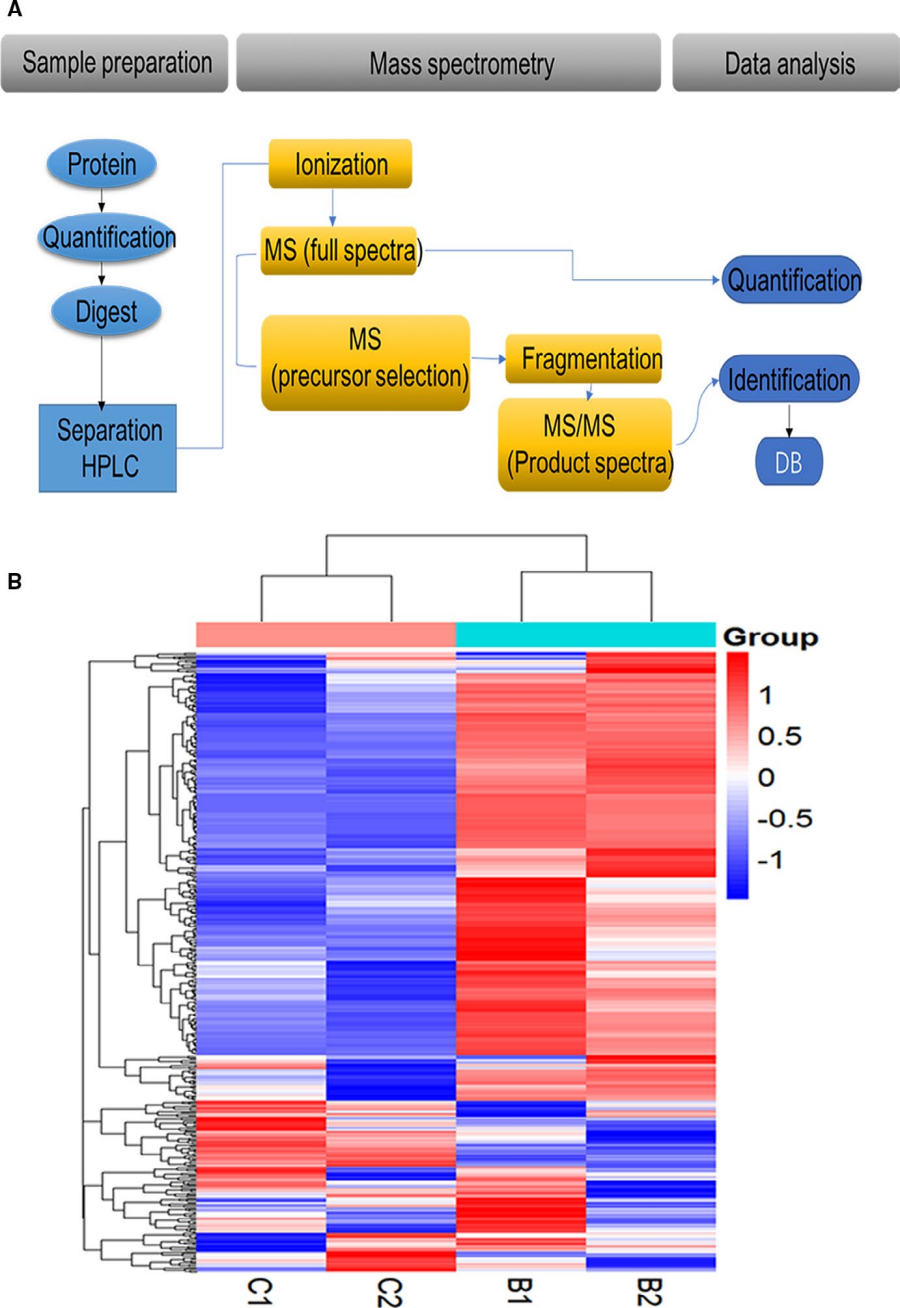
Workflow of label‐free proteomics and heatmap. The workflow was mainly divided into three parts: protein sample preparation, MS analysis, and data analysis (A). MS analysis was performed using MaxQuant software, and the proteins were quantified and identified based on the *Homo sapiens* genome database. The heatmap illustrated the repeatability of the label‐free proteomics technology (B). B1, B2, C1 and C2 represent repeated outcomes of two independent experiments. B represents the patients with POAG combined with cataract (n = 10), and C represents patients with cataract (n = 10)

The process of the label‐free proteomics technology is divided into three main stages: protein sample preparation, MS measurement and data analysis. Ten pooled samples each were available from the POAG combined with cataract group and the cataract group. The protein concentration was 0.15 µg/µL in the POAG combined with cataract group and 0.07 µg/µL in the control group. Proteins in each group were divided into two subgroups, that is, high‐density proteins and low‐density proteins, to identify proteins with a low density. The test was repeated twice using the same method. From the heatmap, we concluded that the repeatability of the results was sufficient and that noticeable differences were observed between the two groups. Red represents up‐regulated proteins and blue represents down‐regulated proteins in the comparison of the POAG and control groups shown in Figure 2.

### 3.3 Data analysis

**FIGURE 3:**
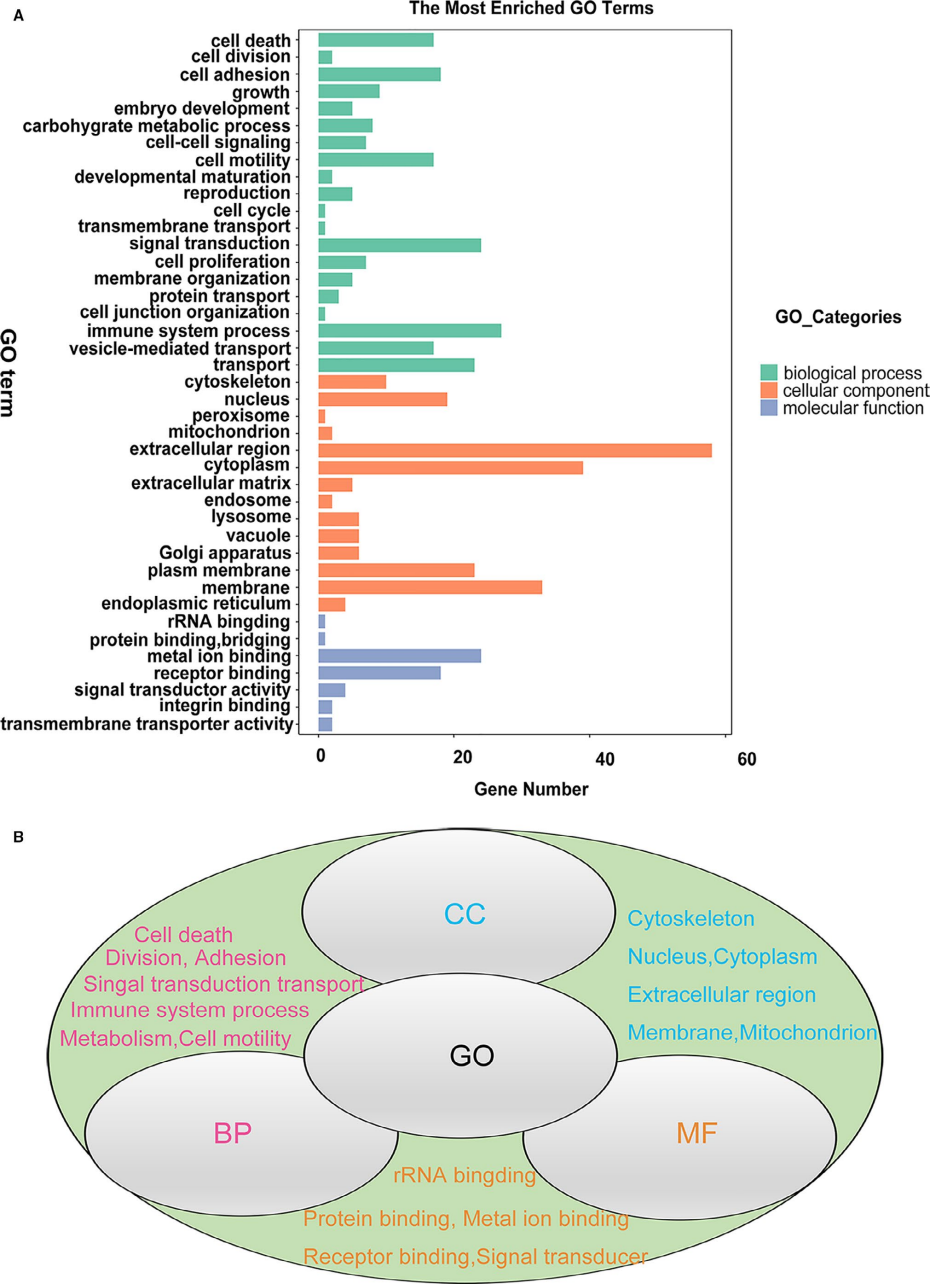
GO analysis of significantly differentially expressed proteins between the POAG combined with cataract group and cataract group. The GO analysis mainly includes three aspects: cellular component (CC), molecular function (MF) and biological process (BP). In the cellular component category, the significantly differentially expressed proteins were mainly enriched in the cytoskeleton and nucleus and mainly located in the extracellular region, cytoplasm, membrane and mitochondrion. In the molecular function category, the significantly differentially expressed proteins were mainly enriched in rRNA binding, protein binding or bridging. Numerous proteins engaged in metal ion binding and receptor binding. Some also participated in signal transduction activity, transmembrane transporter activity and integrin binding. In the biological process category, significantly differentially expressed proteins were enriched in cell death, division, adhesion, growth, metabolism, cell‐to‐cell signal and cell motility. Many more proteins were involved in immune system process, signal transduction and transport

**FIGURE 4:**
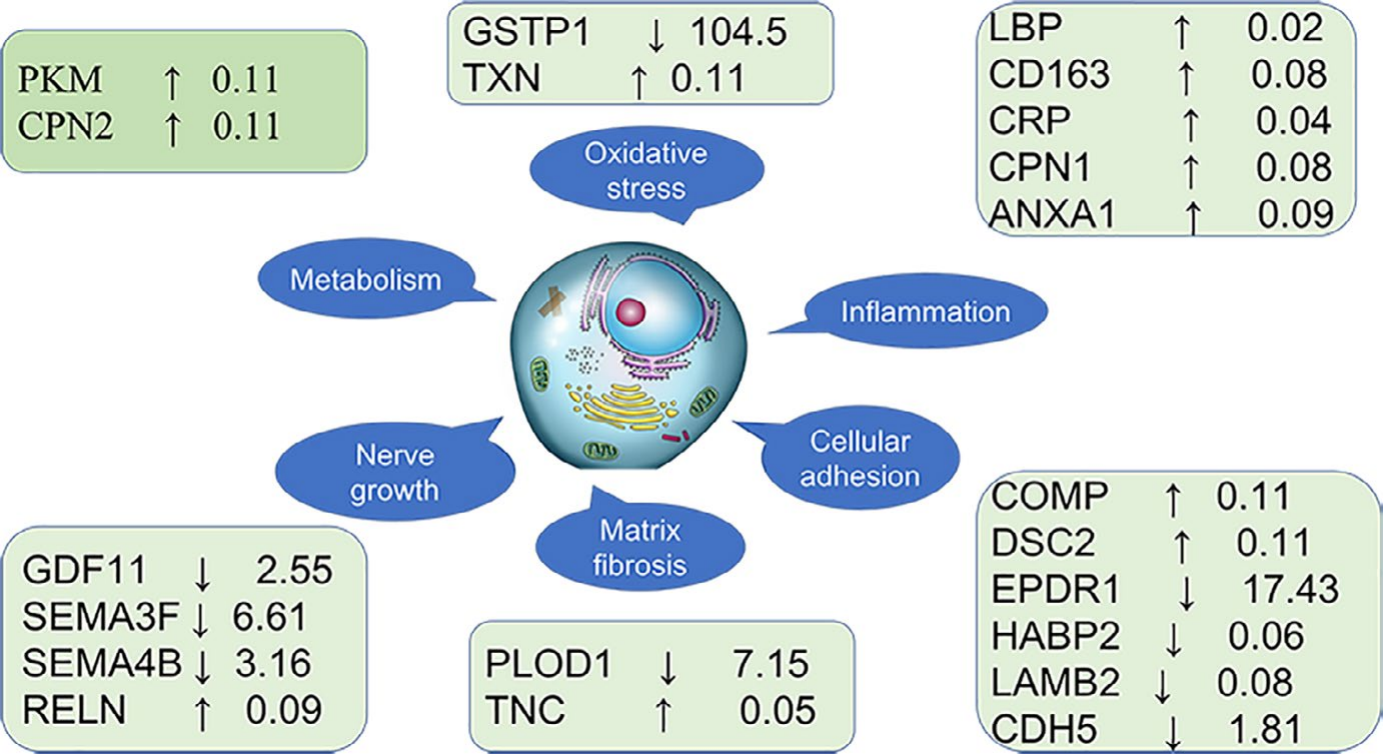
Functions of the main significantly differentially expressed proteins in the comparison of the POAG combined with cataract group and cataract group. Among the significantly differentially expressed proteins, several proteins are listed according to their function and degree of change. These proteins are mainly involved in oxidative stress, inflammation, cellular adhesion, matrix fibrosis, nerve growth and metabolism. Information on specific proteins is listed in the box, including the protein name, up‐ or down‐regulation (arrow) and fold change in the comparison of the POAG combined with cataract group and cataract group

All significantly differentially expressed proteins identified in the LC‐MS analysis are listed in Table 2, which describes the differentially expressed proteins between the POAG combined with cataract group and the control group. Selected interesting proteins are also labelled in Table 2, and their functions are shown in Figure 4. Gene Ontology (GO) analysis revealed the cellular components, molecular functions and biological processes of all differentially expressed proteins using the UniProt website, and the results are shown in Figure 3. KEGG pathway analysis was conducted on the complete data sets of modulated proteins using the KEGG website (http://www.genome.jp/kegg) to highlight possible molecular mechanisms underlying the differential expression of proteins in patients with POAG. Known mutual interactions among differentially expressed proteins were used to construct protein‐protein interaction (PPI) networks with the STRING database.

**TABLE 2:** Up‐ or down‐regulated protein between POAG combined cataract and Cataract

UniProt ID	Gene name	P/C	*P*‐value	Up/down
Q5JWQ4_HUMAN	NRP1	10.078	.034	1
M0QZ24_HUMAN	N/A	8.859	.045	1
J3KQ66_HUMAN	RELN	12.436	.021	1
H7BYX9_HUMAN	PROC	8.865	.045	1
H3BTN5_HUMAN	PKM	33.962	.001	1
G3XAP6_HUMAN	COMP	16.351	.011	1
G3V2V8_HUMAN	NPC2	9.069	.043	1
F5H7V9_HUMAN	TNC	11.756	.024	1
F5H6I0_HUMAN	B2M	12.512	.021	1
F5GZZ9_HUMAN	CD163	26.216	.003	1
E9PHM6_HUMAN	DST	50.572	.000	1
E5RIQ1_HUMAN	LY6E	8.997	.044	1
D6RD58_HUMAN	LECT2	29.651	.002	1
A6QRJ1_HUMAN	ATP6AP1	10.509	.031	1
A0A0U1RR20_HUMAN	PRG4	15.738	.012	1
GNPTG_HUMAN	GNPTG	38.685	.001	1
PCSK1_HUMAN	PCSK1N	31.039	.002	1
LRC4B_HUMAN	LRRC4B	12.710	.020	1
NEUR1_HUMAN	NEU1	12.065	.023	1
SFRP2_HUMAN	SFRP2	13.689	.017	1
SFRP3_HUMAN	FRZB	9.188	.042	1
SNED1_HUMAN	SNED1	24.553	.003	1
CH3L2_HUMAN	CHI3L2	11.897	.024	1
HABP2_HUMAN	HABP2	24.229	.004	1
NID2_HUMAN	NID2	19.434	.007	1
COTL1_HUMAN	COTL1	20.392	.006	1
MGT5A_HUMAN	MGAT5	9.576	.038	1
REG3A_HUMAN	REG3A	8.510	.049	1
DSC2_HUMAN	DSC2	38.833	.001	1
LAMB2_HUMAN	LAMB2	19.989	.006	1
CAPG_HUMAN	CAPG	11.208	.027	1
TKT_HUMAN	TKT	27.104	.003	1
HGFL_HUMAN	MST1	14.122	.016	1
S10A4_HUMAN	S100A4	41.509	.001	1
CPN2_HUMAN	CPN2	14.875	.014	1
LBP_HUMAN	LBP	21.431	.005	1
DESP_HUMAN	DSP	29.863	.002	1
CBPN_HUMAN	CPN1	9.070	.043	1
NID1_HUMAN	NID1	27.549	.002	1
THIO_HUMAN	TXN	12.533	.021	1
LDHB_HUMAN	LDHB	10.637	.031	1
GDN_HUMAN	SERPINE2	30.014	.002	1
ANXA1_HUMAN	ANXA1	22.100	.005	1
CRP_HUMAN	CRP	83.521	.000	1
IGHM_HUMAN	IGHM	10.647	.030	1
FA10_HUMAN	F10	10.586	.031	1
SLIK3_HUMAN	SLITRK3	13.532	.017	1
VTM2B_HUMAN	VSTM2B	9.157	.042	1
X6R5A3_HUMAN	TDP2	0.406	.012	−1
S4R460_HUMAN	IGHV3OR16‐9	0.334	.005	−1
M0QXB0_HUMAN	LSM4	0.584	.048	−1
J3QS39_HUMAN	UBB	0.342	.005	−1
I3L1J2_HUMAN	CDH5	0.076	.000	−1
H0YI30_HUMAN	GDF11	0.396	.011	−1
H0Y332_HUMAN	STXBP5	0.026	.000	−1
G3V164_HUMAN	GRIA4	0.568	.043	−1
F5GZS6_HUMAN	SLC3A2	0.579	.046	−1
C9JPG5_HUMAN	SEMA3F	0.339	.005	−1
C9JP14_HUMAN	ADH7	0.240	.001	−1
B7WNR0_HUMAN	ALB	0.373	.008	−1
B3KTY4_HUMAN	SLITRK2	0.311	.003	−1
A0A0C4DH36_HUMAN	IGHV3‐38	0.527	.033	−1
EPDR1_HUMAN	EPDR1	0.044	.000	−1
NTRI_HUMAN	NTM	0.423	.014	−1
KRT82_HUMAN	KRT82	0.033	.000	−1
SEM4B_HUMAN	SEMA4B	0.361	.007	−1
CBPB2_HUMAN	CPB2	0.532	.034	−1
AEBP1_HUMAN	AEBP1	0.140	.000	−1
SBP1_HUMAN	SELENBP1	0.521	.032	−1
FSTL1_HUMAN	FSTL1	0.576	.045	−1
PLOD1_HUMAN	PLOD1	0.508	.029	−1
KRT85_HUMAN	KRT85	0.057	.000	−1
TPIS_HUMAN	TPI1	0.166	.000	−1
CRBB1_HUMAN	CRYBB1	0.010	.000	−1
CRBA4_HUMAN	CRYBA4	0.294	.003	−1
CRBB2_HUMAN	CRYBB2	0.055	.000	−1
SPB6_HUMAN	SERPINB6	0.487	.025	−1
S10A7_HUMAN	S100A7	0.228	.001	−1
CRBS_HUMAN	CRYGS	0.234	.001	−1
IDS_HUMAN	IDS	0.392	.010	−1
GSTP1_HUMAN	GSTP1	0.430	.015	−1
CRGD_HUMAN	CRYGD	0.364	.007	−1
S10A9_HUMAN	S100A9	0.566	.043	−1
CRBA1_HUMAN	CRYBA1	0.057	.000	−1
K2C6B_HUMAN	KRT6B	0.464	.020	−1
APOB_HUMAN	APOB	0.317	.004	−1
ANGI_HUMAN	ANG	0.564	.042	−1
CRYAA_HUMAN	CRYAA	0.398	.011	−1
HV205_HUMAN	IGHV2‐5	0.151	.000	−1
LV140_HUMAN	IGLV1‐40	0.222	.001	−1
KV133_HUMAN	IGKV1‐33	0.578	.046	−1
CAH1_HUMAN	CA1	0.554	.039	−1
FCN3_HUMAN	FCN3	0.515	.030	−1
LFTY2_HUMAN	LEFTY2	0.366	.007	−1
HV103_HUMAN	IGHV1‐3	0.081	.000	−1
HV226_HUMAN	IGHV2‐26	0.381	.009	−1
LV861_HUMAN	IGLV8‐61	0.529	.033	−1

Note

Green label represents interesting significant proteins. P/C: Primary open‐angle glaucoma combined cataract/Cataract.1: up‐regulated proteins in P, −1: down‐regulated proteins in P.

A total of 610 proteins were detected in the two groups. Ninety‐seven significantly differentially expressed proteins were detected in the AH of patients with POAG combined with cataract compared with patients with cataract (proteins with a corrected *P* < .05 and fold change > 1.5 were considered significantly differentially expressed). Forty‐eight of these proteins were up‐regulated and 49 were down‐regulated. Some of these proteins, such as lipopolysaccharide‐binding proteins (LBP), scavenger receptor cysteine‐rich type 1 protein M130 (CD163), CRP, carboxypeptidase N catalytic chain (CPN1), GSTP1 and annexin A1 (ANXA1), are associated with inflammation. Some proteins, such as thioredoxin (TXN) and GSTP1, function in redox reactions. Other proteins, such as cadherin 5 (CDH5), cartilage oligomeric matrix protein (COMP), desmocollin‐2 (DSC2), mammalian ependymin‐related protein 1 (EPDR1), hyaluronan‐binding protein 2 (HABP2), laminin subunit beta‐2 (LAMB2), procollagen‐lysine, 2‐oxoglutarate 5‐dioxygenase 1 (PLOD1) and tenascin (TNC), are associated with cell adhesion and movement. Some proteins, such as reelin (RELN), semaphorin‐3F (SEMA3F) and semaphorin‐4B (SEMA4B), are associated with nerve growth; metabolism‐related proteins include pyruvate kinase (PKM) and carboxypeptidase N subunit 2 (CPN2).

### 3.4 GO, KEGG and PPI analyses

**FIGURE 5:**
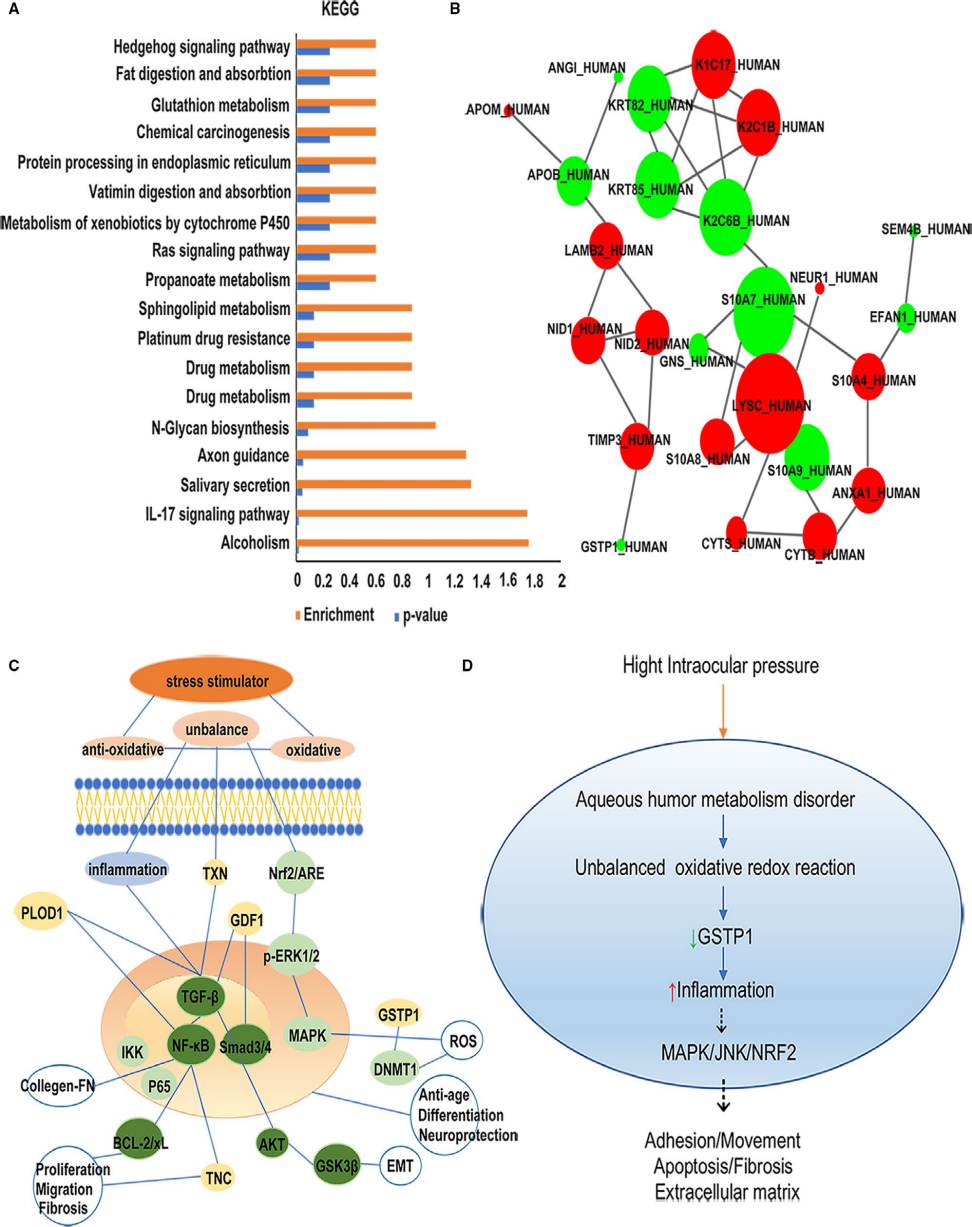
KEGG, PPI and signalling pathway analyses. The main enriched signalling pathways were the IL‐17 signalling pathway, the axon guidance signalling pathway and drug metabolism signalling pathway. A, PPI networks reflect the interactions of proteins with other proteins. In the comparison of the POAG combined with cataract group with the cataract group, red represents up‐regulated proteins, and green represents down‐regulated proteins. The size of the circle represents the amount of the related protein (B). The signalling pathways were acquired from our data analysis and previous articles (C and D)

GO analysis mainly evaluates three aspects: cell components, molecular functions and biological processes. The differentially expressed proteins were enriched mainly in the cytoskeleton region, nucleus, cytoplasm, extracellular region, cell membrane, mitochondrial endoplasmic reticulum and vesicles. The main cellular functions of all differentially expressed proteins were rRNA binding, protein binding, metal ion binding, receptor binding, signal transducer and transmembrane transport. All differentially expressed proteins were enriched mainly in the biological processes of immune response, cell death, division, adhesion, movement, growth and development and signal transduction transport (Figure 4). We obtained signalling pathways from the KEGG website by analysing all significantly differentially expressed proteins. These signalling pathways were ranked according to the p‐value and enrichment. The IL‐17 signalling pathway plays a vital role in the inflammatory response. Glutathione metabolism also exerts an indispensable effect on inhibiting oxidative stress. The signalling pathway and metabolism of xenobiotics by cytochrome P450 have been verified to be involved in the pathology of congenial glaucoma. Most signalling pathways are related to metabolism, such as drug metabolism, glutathione metabolism, propanoate metabolism and sphingolipid metabolism. Axon guidance is a subfield of neural development concerning the process by which neurons send out axons to reach the correct targets. The Hedgehog signalling pathway participates in the differentiation of embryonic cells. The Ras signalling pathway is very important in regulating cell proliferation, survival, growth, migration, and differentiation and cytoskeletal dynamics. These signalling pathways cover a broad range of molecular biological processes. S100A8 in the IL‐17 signalling pathway was up‐regulated in patients with POAG combined with cataract. The expression of GSTP1 in the glutathione metabolism signal pathway was down‐regulated. PPI networks explain the mutual connections between different proteins. We input significantly differentially expressed proteins into the STRING website and constructed the network with Cytoscape software. Red represents up‐regulated proteins, and green represents down‐regulated proteins. These differentially expressed proteins are involved mainly in inflammation, oxidative stress, metabolism and remodelling of ECM proteins. Among these differentially expressed proteins, GSTP1 regulates the activity of glutathione and prevents neurodegeneration. Tissue inhibitor of metalloproteinase 3 (TIMP3) may play a role in tissue remodelling induced by acute stimulation. Nidogen 1 (NID1) and nidogen 2 (NID2) are involved in the formation of tight junctions of the basement membrane and the transmission of ECM signals. LAMB2 is involved in the adhesion, migration and reconstruction of cellular tissues. Apolipoprotein B (APOB) and apolipoprotein M (APOM) are involved in lipid metabolism. Many other proteins, such as S100‐A7 protein (S10A7), S100‐A8 protein (S10A8), S100‐A9 protein (S10A9), S100‐A4 protein (S10A4) and ANXA1, are involved in the inflammatory response. Keratin‐82 (KRT82), keratin‐85 (KRT85), cytokeratin17 (K1C17), cytokeratin‐1B (K2C1B) and cytokeratin‐6B (K2C6B) are related to the formation of keratin and keratinization of the epithelium (Figure 5).

### 3.5 Protein validation with ELISA

**FIGURE 6:**
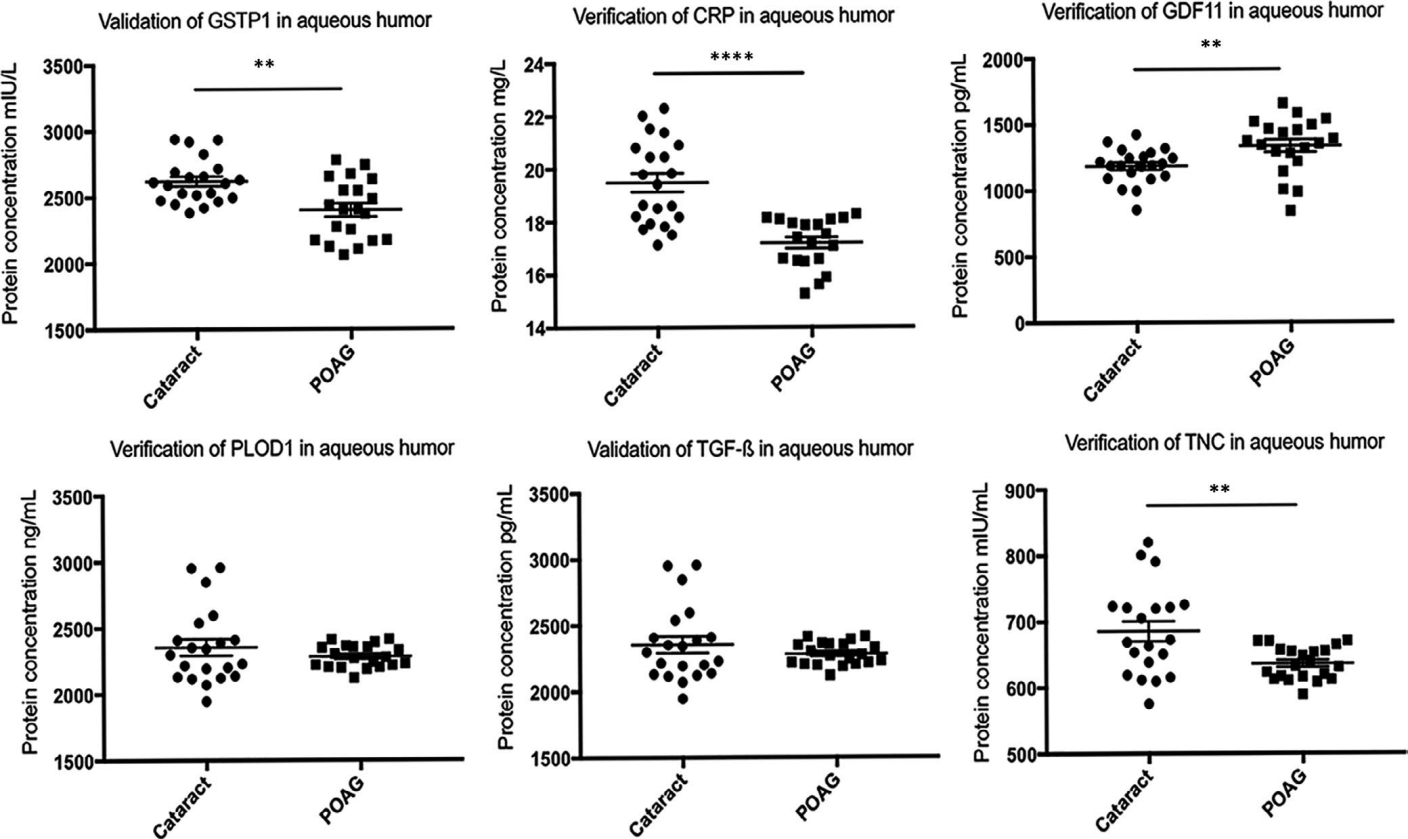
Verification of differentially expressed proteins in the AH of patients with POAG combined with cataract and patients with cataract using ELISAs. Clinical information of the patients is listed in Table 3. The levels of the GSTP1, CRP, GDF11, PLOD1, TGF‐ß and TNC proteins were verified. **P < .01 and ****P < .0001

AH was collected again from patients with POAG combined with cataract and control patients. The patients’ clinical information is shown in Table 3. Figure 6 shows the results of the ELISA verification. Among the proteins, the levels of GSTP1, CRP and TNC were reduced in the AH samples from the POAG combined with cataract group, the GDF11 level was increased in the POAG combined with cataract group, and no significant differences in PLOD1 and TGF‐ß levels were observed between the two groups. The GSTP1 expression level was consistent with the proteomic data (Figure 6).

**TABLE 3:** Demographic and clinical characteristics of Cataract, POAG combined cataract subjects

Characteristics	POAG	Cataract	*P*‐value	Significance
Subjects, n	20	21		
Male/female	10/10	12/9	>.05	ns
Age, y (mean ± SD)	66.00 ± 10.00	69.00 ± 10.00	>.05	ns
Cup/disc ratio (mean ± SD)	0.80 ± 0.20	0.30 ± 0.20	.0001	***
IOP (mean ± SD)	33.95 ± 1.20	15.59 ± 0.60	<.0001	****
Axial length (mean ± SD) mm	23.93 ± 0.20	23.23 ± 0.49	>.05	ns
Corneal thickness (mean ± SD) µm	510 ± 7.02	524.1 ± 6.84	>.05	ns
ACD (mean ± SD) mm	3.01 ± 0.03	2.98 ± 0.18	>.05	ns
BCVA (mean ± SD)	0.20 ± 0.10	0.40 ± 0.30	>.05	ns
Other disease history	–	–	–	

Note

Statistical analysis: Nonparametric *t* test (***P < .001; ****P < .0001; ns: no significant difference).

Abbreviations: ACD, anterior chamber depth; BCVA, best corrected visual acuity; IOP, intraocular pressure; SD, standard deviation.

## 4 DISCUSSION

In recent years, the application of omics technology in the field of biomedical research has become increasingly widespread, which has enhanced the data output capability of life science research. Proteomics is an important approach used to explore differentially expressed proteins related to various diseases and has been widely applied in glaucoma, cataract, corneal lesions, macular degeneration, and uveitis and other diseases.^25^, ^26^, ^27^, ^28^, ^29^, ^30^, ^31^ Compared with labelled protein profiling, unlabelled MS has the advantages of a lack of limitation on the sample size, the low cost of isotope labelling and the ability to detect a wide range of proteins.^32^

The dynamic balance of the AH is closely related to IOP, which is an important risk factor for POAG. Changes in AH components also reflect tissue metabolism and pathological processes in the anterior segment of the eye. At the same time, because AH is relatively easy to obtain and convenient to store, it is a better sample source for exploring glaucoma. A review of the proteomic data from studies focused on the pathogenesis of POAG that have been conducted over the last decade indicated that studies employing diverse proteomics technologies, such as Sequential Window Acquisition of all Theoretical Mass Spectra (SWATH),^26^ label‐free proteomics,^25^ antibody microarray analysis,^33^ RT2 Custom Profiler PCR Array analysis,^34^, ^35^ liquid chromatography‐mass spectrometry (LC‐MS)^36^ and LC‐MS/MS,^37^ to analyse the AH of patients with glaucoma showed that proteins associated with oxidative stress and inflammation are significantly differentially expressed (Table 4).

**TABLE 4:** Comparison aqueous humour proteins expression of POAG in previous publications

PMID	Mean age (POAG/cataract)	Nation/ethnicity	Tissue	Size (POAG/cataract)	Method	Significant protein	Similar gene name	Oxidative stress‐related proteins	Protein function
Our results	73/72	Tianjin, Chinese	HAH	10/10	LFQ	97	GSTP1	GSTP1, TXN	Inflammation, redox reaction, cell adhesion and movement, nerve growth, metabolism
27788204	75/78	Aarau, Switzerland	HAH	5/5	SWATH	87	TKT, GSTP1, CRYAA	PRDX1, CAT	Cholesterol‐related, inflammatory, metabolic, antioxidant, proteolysis‐related
26265374	58/61	Shanghai, Chinese	HAH	6/6	iTRAQ	262	SERPING1,NPC2,FBLN1,SPINK4,CA1,FCGR3A,APOF,RELN,DSP,FCGR3A,PCSK1N,IGHM,CRYBB1,CRYBA4	–	Catalytic, complement, enzymatic, signalling, structure, transporting
27193151	77/68	Moscow, Russia	HAH	7/11	LFQ	36	ALB, SERPINF1 (PEDF)	–	Lipoproteins, immunoglobulins, carrier proteins, neurotrophic, development of the neural system
29332228	56/55	New Delhi, India	HAH	9/9	LC‐MS/MS	97	GSTP1	SOD, GPx, TRAP	Tissue and vascular remodelling, immune response, blood coagulation Antioxidant activity
29847670	66/65	Augusta, America	HAH	15/32	LC‐MS	33	NPC2	–	Signalling, immune response, molecular transporting, lipid metabolism
22974818	75/73	Genoa, Italy	HAH	10/10	Antibody microarray	4	SOD1/2, GST1 NOS2, GS	GS, NOS, SOD, GST	Aqueous humour oxidative stress proteomic levels in primary open‐angle glaucoma
20666514	75/73	Caucasian	HAH	10/14	Antibody microarray	30	CDH5	PRKCE, PRKCD, PRKACA, PRKCQ, NOS2, SOD1/2, MGST1DNCL1	Oxidative damage, mitochondrial damage, neural degeneration and apoptosis
30994369	60/63	Chennai, India	HAH	90/78	LC‐MS/MS	87	IGHV3OR16‐9	–	Complement and coagulation cascade, regulation of wound healing, inflammatory response and extracellular matrix organization
32246983	74/71	Florida, America	HAH	23/35	NMR, IROA	5	–	–	Metabolism: lysine, arginine, cysteine and glycine

Based on a large number of reports related to POAG research combined with the potential differentially expressed proteins identified in our current proteomic data set, we have been suggested that both inflammation and oxidative stress reactions are involved in the pathological changes in the homeostasis of the AH microenvironment, leading to metabolic dysfunction in the anterior chamber tissue. This dysfunction is specifically manifested as ECM disorders, leading to insufficient drainage of the AH and ultimately to an elevated IOP. AH samples were collected from separate patients with POAG combined with cataract and control patients to verify the changes in the levels of inflammatory factors, redox response‐related proteins and ECM proteins in the AH using ELISA and to confirm our hypothesis. We subsequently selected six proteins (GSTP1, GDF11, CRP, PLOD1, TGF‐ß and TNC) that are closely related to the pathogenesis of POAG for subsequent verification using ELISA to exclude the possibility of false positives. As shown in the present study, the change in GSTP1 levels measured by ELISA was consistent with the protein profile; in other words, GSTP1 was expressed at significantly lower levels in patients with POAG than in control individuals. Moreover, the opposite results were obtained for the TNC, GDF11 and CRP levels, and no difference in the PLOD1 and TGF‐ß levels was detected by ELISA. The potential explanations for these inconsistencies include individual differences, the small sample size and shifts in the protein profile.

GSTP1, a member of the glutathione S‐transferase (GST) family, catalyses the binding of many hydrophobic and electrophilic compounds to reduced glutathione and has a strong antioxidant capacity. Silencing GSTP1 in patients with chronic obstructive pulmonary disease (COPD) increases reactive oxygen species (ROS) production and DNA damage in cells.^38^ Loss of GSTP1 expression in human prostate cells increases DNA damage caused by oxidative stress.^39^ Notably, (a) GSTP is a gene downstream of the nuclear factor erythropoietin 2‐related factor 2 (Nrf2)‐antioxidant response element (ARE)/electrophilic response element (EpRE) transcription pathway.^40^ (b) GSTP1 reduces ROS expression and apoptosis induced by oxidative stress through the Nrf2‐extracellular signal‐regulated protein kinase1/2 (ERK1/2)‐mitogen‐activated protein kinase (MAPK) pathway and exerts a neuroprotective effect.^41^, ^42^ (c) Oxidative stress may increase methylation of the GSTP1 and thioredoxin reductase 2 (TXNRD2) gene promoters by up‐regulating DNA methyltransferase 1 (DNMT1). Increased methylation decreases the transcription of GSTP1 and TXNRD2. Oxidative stress interacts with gene methylation to form a vicious cycle.^43^ The redox imbalance promotes the secretion of inflammatory factors and contributes to other reactions induced by inflammation.^44^, ^45^ GST is a multigene family with multiple enzymes that play different roles in anti‐oxidation, detoxification and elimination of xenobiotics, including carcinogens, oxidants, toxins and drugs. GSTP1 Ile 105 Val polymorphism results in an absence of their enzyme activity. A meta‐analysis on various GST mutations discovered that a polymorphism in the GSTP1 gene was significantly correlated with increased POAG risk in a Caucasian population.^46^ The GSTM1 null/GSTP1, Ile/Val or Val/Val genotypes were associated with increased IOP and more advanced defect of the right eye optic nerve and visual field.^47^ The frequency of the GSTT1 and GSTP1 mutation was not statistically different between POAG patients and healthy controls group based on genomic DNA from peripheral blood.^48^ In conclusion, the relationship between GSTP1 and POAG remains undetermined. Further, the above‐mentioned studies made their conclusions based on gene level from patients’ blood. Our research found that GSTP1 protein expression was decreased in aqueous humour of POAG patients. So, we have reason to believe that GSTP1 may be a possible biomarker in POAG pathogenesis.

CRP has been widely used in clinical practice as an indicator of inflammation. Changes in its level suggest the existence of an inflammatory reaction in the AH microenvironment.^49^ According to previous studies, inflammation is associated with changes in the ECM of patients with glaucoma.^50^, ^51^, ^52^ UniProt data show that GDF11, also called BMP11, regulates cell development and mutations to influence ganglion cell formation and eye morphogenesis.^53^ The GDF11 gene encodes a secreted ligand of the TGF‐β superfamily. Ligands of this family bind various TGF‐β receptors, leading to the recruitment and activation of SMAD family transcription factors that regulate gene expression.^54^ The GDF11 level in the body decreases with increasing age, and its use in individuals with renal ischaemia‐reperfusion injury promotes kidney repair. By activating the ERK1/2 pathway in vitro, the addition of recombinant GDF11 to primary renal epithelial cells promotes the regeneration of luminal cells.^55^ A high IOP may lead to an insufficient blood supply to the retinal layer and a disturbance in optic nerve metabolism, which may be related to the disruption in GDF11 expression. Although the data for CRP and GDF11 from our ELISAs were not supported by our Nano‐HPLC‐MS data, this study provides a potential target or a meaningful insight into the association between POAG and GDF11, as well as CRP regulation, which is also of considerable significance.

Compared with a single multi‐facility study, we further examined the literatures in search for proteins with functions similar to those we found. They analysed aqueous humour, used different testing methods, and studied those of different races. From these studies, we noted the differentially expressed proteins in POAG related to oxidative stress. The GST family was also implicated in these studies. Other proteins, such as those involved in neuronal protection, have also attracted our attention. We believe that inflammation, oxidative stress and neuroprotection are candidate signalling pathways for future therapeutic development.

All patients were enrolled in strict accordance with the inclusion criteria to reduce bias. Based on clinical reality and medical ethics, we were unable to collect AH samples from healthy people at the clinic. Therefore, in this experiment, patients with POAG combined with cataract were enrolled as the study group and compared with patients with cataract alone as the control group. We must not completely ignore the changes in the AH of the cataract group compared with the healthy group. In addition, the medication status of the patients in the POAG group should not be ignored. Different patients took different medications but generally used ß‐adrenergic receptor agonists and neuroprotective drugs. Drug interference cannot be ruled out, mainly for reasons of patient safety.

An imbalance in redox reactions is caused by various factors, leading to increased inflammation. Other factors promote changes in the levels of ECM proteins and increases in the ECM cross‐linking of TM cells, resulting in blocked AH release, increased IOP and retinal nerve disruption, which eventually lead to the onset or progression of POAG. GSTP1, a redox‐related protein, is expected to become a target for preventing the onset of or treating glaucoma. Our follow‐up study will focus on the biological functions of GSTP1 in TM cells under oxidative stress.

## CONFLICTS OF INTEREST

The authors confirm that no conflicts of interest exist.

## AUTHOR CONTRIBUTION

**Aihua Liu:** Funding acquisition (equal); Resources (equal); Visualization (equal); Writing‐original draft (equal). **Liming Wang:** Resources (equal); Writing‐original draft (equal); Writing‐review & editing (equal). **Qiang Feng:** Data curation (equal); Supervision (equal). **Dandan Zhang:** Data curation (equal); Resources (equal). **Kexi Chen:** Data curation (equal); Resources (equal). **Guli Humaer Yiming:** Data curation (equal); Resources (equal). **Qiong Wang:** Methodology (equal); Software (equal). **Yaru Hong:** Methodology (equal); Resources (equal). **Amy Whelchel:** Formal analysis (equal); Investigation (equal). **Xiaomin Zhang:** Resources (equal); Supervision (equal). **Xiaorong Li:** Conceptualization (equal); Funding acquisition (equal); Supervision (equal); Visualization (equal); Writing‐review & editing (equal). **Lijie Dong:** Project administration (equal); Supervision (equal); Visualization (equal); Writing‐review & editing (equal).

## Data Availability

The data that support the findings of this study are available from the corresponding author upon reasonable request.
